# Rolling ball sifting algorithm for the augmented visual inspection of carotid bruit auscultation

**DOI:** 10.1038/srep30179

**Published:** 2016-07-25

**Authors:** Adam Huang, Chung-Wei Lee, Hon-Man Liu

**Affiliations:** 1 Research Center for Adaptive Data Analysis, National Central University, Jhongli, 32001, Taiwan; 2 Department of Medical Imaging, National Taiwan University Hospital, Taipei, 100, Taiwan; 3 Department of Radiology, College of Medicine, National Taiwan University, Taipei, 100, Taiwan

## Abstract

Carotid bruits are systolic sounds associated with turbulent blood flow through atherosclerotic stenosis in the neck. They are audible intermittent high-frequency (above 200 Hz) sounds mixed with background noise and transmitted low-frequency (below 100 Hz) heart sounds that wax and wane periodically. It is a nontrivial task to extract both bruits and heart sounds with high fidelity for further computer-aided auscultation and diagnosis. In this paper we propose a rolling ball sifting algorithm that is capable to filter signals with a sharper frequency selectivity mechanism in the time domain. By rolling two balls (one above and one below the signal) of a suitable radius, the balls are large enough to roll over bruits and yet small enough to ride on heart sound waveforms. The high-frequency bruits can then be extracted according to a tangibility criterion by using the local extrema touched by the balls. Similarly, the low-frequency heart sounds can be acquired by a larger radius. By visualizing the periodicity information of both the extracted heart sounds and bruits, the proposed visual inspection method can potentially improve carotid bruit diagnosis accuracy.

There are often no symptoms of carotid artery disease such as stenosis (narrowing) until a transient ischaemic attack or stroke occurs. Listening for a bruit in the neck is a simple, safe, and inexpensive way to screen for stenosis of the carotid artery. A carotid bruit is an abnormal systolic sound associated with turbulent blood flow through atherosclerotic stenosis in the carotid artery^1^,^2^. Therefore, carotid auscultation was once considered a cost-effective and noninvasive diagnostic tool for the detection of carotid artery stenosis and the estimation of stroke risk for asymptomatic patients^3^,^4^. However, the effectiveness of carotid bruit auscultation has been deemed uncertain because not every symptomatic carotid artery produces bruits^5^,^6^ and most carotid bruits found in younger or asymptomatic patients are not related to any disease^1^,^7^,^8^. Finding a bruit in an asymptomatic patient can cause a lot of anxiety that outweighs the potential benefits^2^. The US Preventive Services Task Force and the Canadian Task Force have both recommended against routine auscultation of carotid arteries in asymptomatic patients^9^,^10^. Nevertheless, in two recent meta-analyses, the presence of a carotid bruit significantly increased the risk of myocardial infarction and cardiovascular death^11^, and may increase the risk of cerebrovascular diseases^12^. In short, auscultation of the neck for carotid bruits remains a useful part in the diagnosis and management of patients with suspected transient ischaemic attack, stroke, or cardiovascular diseases^2^,^8^.

Typically carotid bruits are best heard using the bell of the stethoscope where the skin acts as a diaphragm with the patients holding their breath^8^. The bruits recorded by an electronic/digital stethoscope in the neck are audible intermittent high-frequency (above 200 Hz)^13^ sounds mixed with background noise and transmitted low-frequency heart sounds^3^ that wax and wane periodically. Fourier-based sound spectra analysis such as carotid phonoangiography^14^, quantitative spectral phonoangiography^15^, and more advanced color-shaded spectrograph^13^ have achieved different levels of success^8^. In a clinical setting, for patients with suspected stroke or cardiovascular diseases, breathing quietly may be a better alternative if holding breath is impossible or painful. However, the Fourier spectrum can only give meaningful interpretation to linear and stationary processes^16^, the additional breathing sound is a great challenge to the existing methods to extract “high-fidelity” bruits and transmitted heart sounds for further analysis. Among modern signal processing methods, the empirical mode decomposition (EMD) approach^17^ stands out as a good candidate to resolve the problem as the method has shown great strength in extracting nonlinear, nonstationary signals through sifting local extrema in the time domain. The proposed rolling ball sifting algorithm^18^ has been derived from the original EMD approach with an additional “ball” tangibility criterion for selecting the extrema of similar oscillating timescale to improve its frequency selectivity mechanism.

Intuitively, our rolling ball sifting algorithm works by rolling two balls (one above and one below) of a chosen radius simultaneously along the oscillating data curve as demonstrated in Fig. 1 (also see Supplementary Information for the software implementation). The data points touched by the ball above and the ball below the curve form the upper and lower envelopes, respectively according to the chosen radii. The raw rolling ball envelopes (red and green lines in Fig. 1b) of a bruit are well-defined in the field of computational geometry as alpha shapes^19^,^20^. It has been proved that the alpha shape of a set of data points is a subgraph of the Delaunay triangulation (DT) of the set of data points^19^. Therefore, the raw rolling ball envelopes in Fig. 1b can be found very efficiently by DT^18^ (a partial DT is given in Fig. 1c). For constructing smoother rolling ball envelopes, we need to sift out the local maxima only touched by the upper ball and the local minima only touched by the lower ball respectively. Last, taking a monotone piecewise cubic spline interpolation of the sifted maxima and minima to form the smooth upper and lower envelopes (Fig. 1d), we are able to define a local zero for the extraction of high-frequency signals such as bruits very effectively as shown in Fig. 1e.

Here we first demonstrate that cardiac cycle and heart rate can be derived from the proposed rolling ball envelopes by using a large radius. The cardiac cycle is important because it serves as the landmark to locate systolic bruits. Second, we use a small rolling ball radius to detect bruits over 200 Hz. Third, a Fourier-based high pass filter is also used to detect bruits as a second opinion as well as a comparison. Last, the periodicity information of the bruits by rolling ball and high pass filter is computed and compared to the heart rate for identifying true bruits from breathing noises. We present a test of 96 carotid sound datasets (from 48 patients’ right and left carotid arteries). The agreement between the results of the rolling ball sifting algorithm and high pass filter is high. The rolling ball sifting algorithm complements the high pass filter with a sharper frequency discrimination capability of indicating the existence of bruits.

## Results

### Cardiac Cycle and Heart Rate Detection

In evaluation of bruits, an important aspect is their relationship to the cardiac cycle. The reason is that true bruits are more likely to occur in the systolic cycle rather than in a random pattern. Therefore, high-frequency intermittent noise patterns repetitively synchronizing with the cardiac cycle are adopted as the key indicator for detecting carotid bruits in this paper.

**Figure 1:**
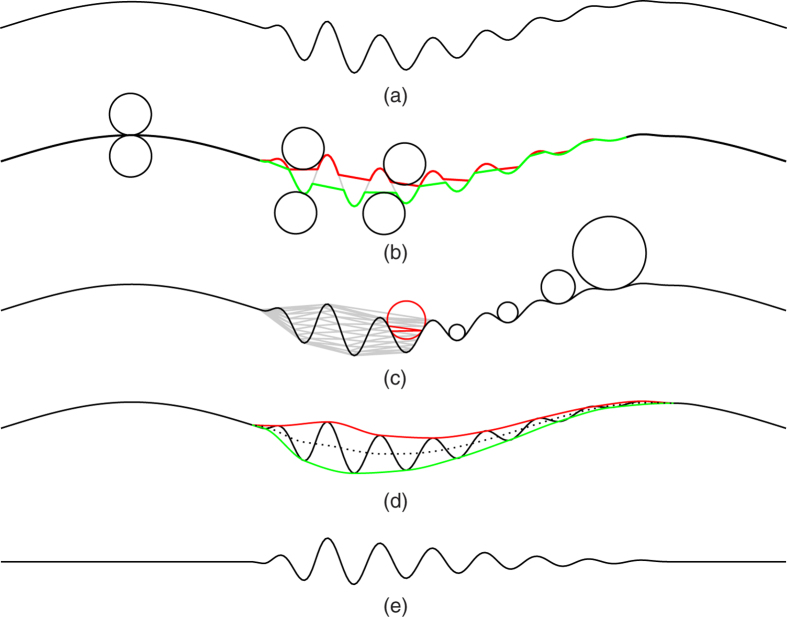
A rolling ball sifting example. (**a**) A simulated carotid bruit signal. (**b**) One ball rolling above and the other below the signal to derive “alpha shapes” as the upper (red) and lower (green) envelopes of the bruit. (**c**) A partial DT illustrates a circumcircle (red) for computing the tangent radius information. Four circles on the right show how the amplitude affects the tangent radius. (**d**) The non-overlaid envelope segments found in (**b**) and their neighbor oscillating waves with small amplitude are merged and inflated. (**e**) The bruit can be extracted by subtracting the mean (dotted line in (**d**)) of the upper and lower envelopes from the signal.

**Figure 2:**
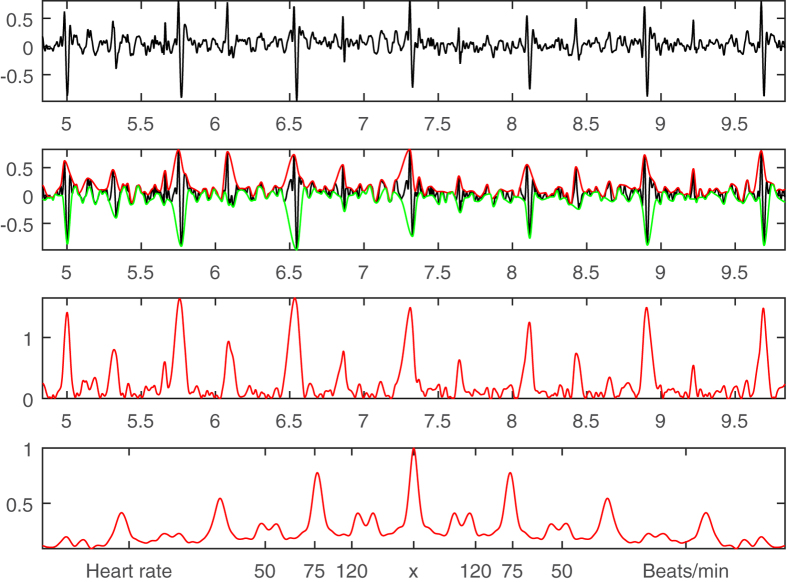
Cardiac cycle and heart rate detection. **Row 1:** A 5-second-long carotid sound signal. **Row 2:** Rolling ball envelopes with a cutoff frequency of 5 Hz. **Row 3:** Cardiac cycle abstracted as the impulse magnitude variation derived from the difference between the upper and lower envelopes (red and green lines in Row 2). **Row 4:** Autocorrelation of the red impulses (Row 3) for deriving the heart rate of 75 beats/min.

Observing our carotid sound dataset, we find that at least one of the two major impulses associated with the first and second heart sounds are broadly visible features (especially the first heart sound) in identifying the cardiac cycle (Fig. 2). These impulse-like oscillations roughly range from 10 to 100 Hz. Therefore, using a rolling ball radius that is able to generate the upper and lower envelopes of oscillating waves above 5 Hz is a reasonable choice to find the impulse magnitude variation of transmitted heart sounds. The rolling ball radius of a cutoff frequency of 5 Hz can be computed by^18^


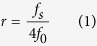


where *r* is the rolling ball radius, *f*_*s*_ and *f*_0_ are the sampling and cutoff frequencies respectively. The radius *r* is a quarter of *f*_*s*_/*f*_0_ (instead of a half) because the rolling ball’s diameter is equivalent to the distance between zero-crossings at the cutoff frequency (Fig. 1c), which is 50 in the base unit of sampling point for *f*_*s*_ = 4000 Hz. The signal’s amplitude (maximal value is 1 in its original WAVE format) is rescaled by a factor of 50 to allow rolling ball function properly for small amplitude signals such as those shown in Fig. 1c. The upper and lower envelopes (Fig. 2, Row 2) and their difference (Fig. 2, Row 3) appear to be a more regular cardiac pulsation shape that allows further processing for the derivation of heart rate. Because the rolling ball processed pulses have a relatively steadier rhythm than the original signal, the heart rate can be easily computed by using the autocorrelation method^21^.

The correlation between two waveforms is a measure of their similarity. The autocorrelation function is the correlation of a waveform with itself as


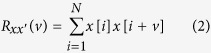


where *x*′ is a copy of itself with a time shift *v*; that is to say, the waveforms are compared at different time intervals, and their “sameness” is calculated at each interval. The central peak shown on Row 4 in Fig. 2 is the correlation of the signal *x* to itself (*v* = 0) and it is normalized to be 1. The maximal peak next to the central peak represents the resultant heart rate, which is about 75 beats per minute.

### Bruit Detection by Rolling Ball Sifting

**Figure 3:**
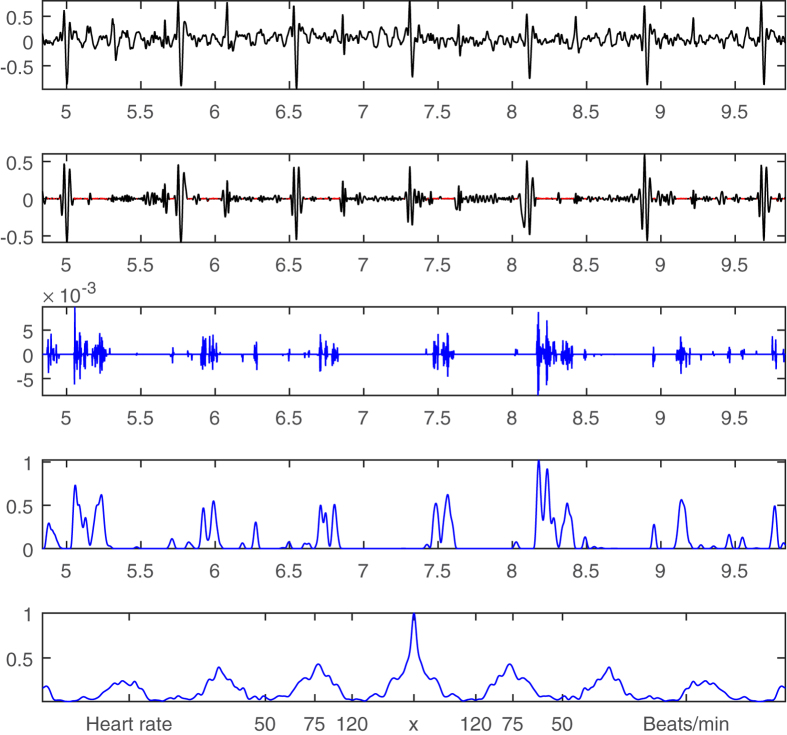
Bruit detection by rolling ball sifting. **Row 1:** A 5-second-long carotid sound signal (same as Fig. 2). **Row 2:** IMF1 of the carotid sound in black with the upper rolling ball envelopes of the intermittent bruits with a cutoff frequency of 200 Hz in red. **Row 3:** Extracted bruits magnified 100 times in blue. **Row 4:** The convolution results of the extracted bruits in Row 3 by a Hanning window of 0.05 seconds. **Row 5:** Autocorrelation of the blue bruit signals (Row 4) for deriving their periodicity information.

As the bruits measured by a stethoscope can have very small amplitudes as compared to the transmitted heart sounds (Fig. 3), the carotid sound signal is first processed by the original EMD algorithm to extract the highest frequency component for the EMD’s high sensitivity in detecting faint signals. The strength of the original EMD’s sifting method lies in its capability to identify any unspecified intrinsic oscillatory mode that rides on a local zero defined as the mean of some upper and lower envelopes. In the classical EMD approach, these upper and lower envelopes are derived by interpolating every local maxima and minima (no ball tangibility criterion involved) with cubic splines respectively. The procedure is usually repeated a few times until the local zero converges and the oscillatory mode riding on the local zero satisfies all the requirements of intrinsic mode functions (IMFs). The highest frequency component is known as the first intrinsic mode function (IMF1) in the field of Hilbert-Huang Transform (HHT)^17^. IMF1 is then processed by using the proposed rolling ball sifting algorithm with a cutoff frequency of 200 Hz. The rolling ball radius is 5 in the unit of sampling points according to Eq. 1. IMF1 and bruits’ upper rolling ball envelopes are shown on Row 2 in Fig. 3. The rolling ball sifted bruits are magnified 100 times and shown on Row 3 in Fig. 3.

Qualitatively, the extracted bruits shown on Row 3 in Fig. 3 demonstrate a clear periodic pattern along the cardiac cycle shown in Fig. 2. However, in order to derive a more quantitative description, the envelopes of the raw bruits signals (Fig. 3, Row 3) are convolved with a Hanning window of 0.05 sec to form a more regular impulsive form (Fig. 3, Row 4) that can be computed by the autocorrelation method (Eq. 2). The resultant periodicity is shown on Row 5 in Fig. 3 with a similar peak at the value of 75 beats/min as the heart rate shown in Fig. 2. This periodicity agreement indicates that these extracted high-frequency signals are very likely to be true bruits.

### Bruit Detection by High Pass Filtering

**Figure 4:**
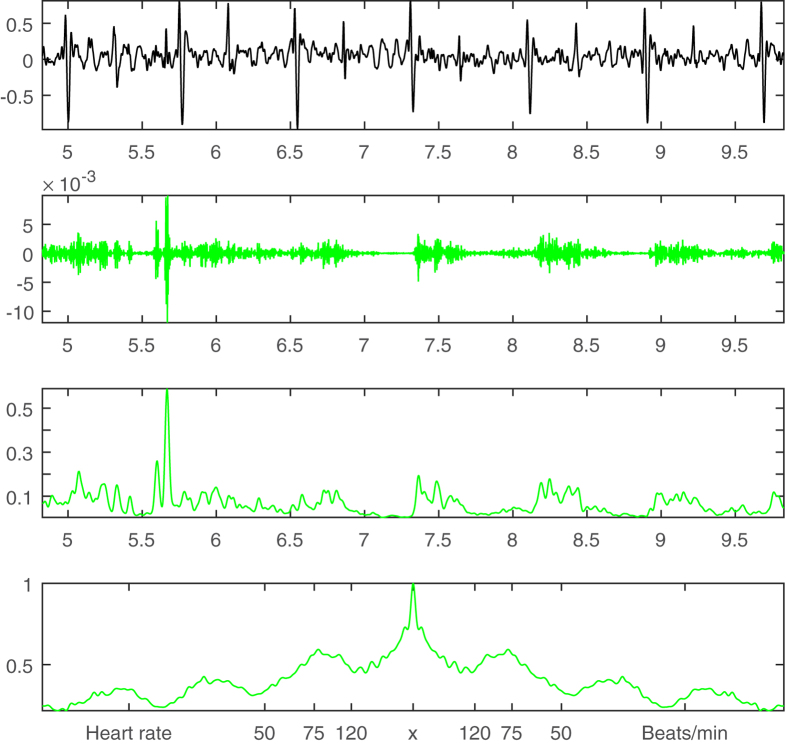
Bruit detection by high pass filtering. **Row 1:** A 5-second-long carotid sound signal (same as Fig. 2). **Row 2:** Fourier-based high pass (>200 Hz) filtered bruits magnified 100 times in green. **Row 3:** The convolution results of the extracted bruits on Row 2 by a Hanning window of 0.05 seconds. **Row 4:** Autocorrelation of the green bruit signals (Row 3) for deriving their periodicity information.

**Figure 5:**
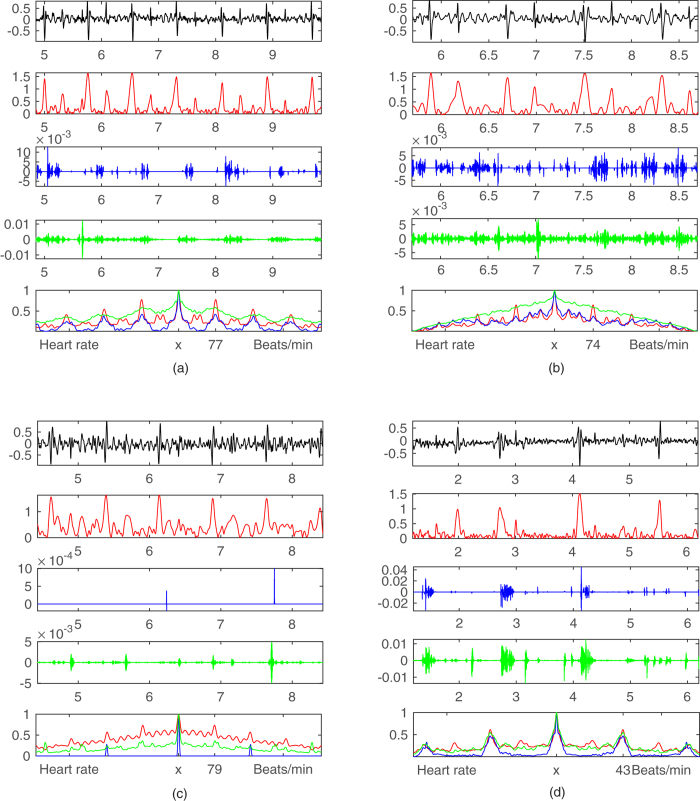
Augmented visual inspection system: with the original signal (black), extracted heart sound pattern (red), rolling ball sifted bruits (blue), high pass filtered bruits (green), and their periodicity by autocorrelation. (**a**) True bruits have both rolling ball and high pass results agree with the heart sound pattern. (**b**) Only the rolling ball sifted bruits agree with the heart sound pattern. (**c**) Bruits are only detected by the high pass filter. (**d**) A complete occluded carotid artery has visible bruits.

For the purpose of comparison and as a second opinion, the same bruit signal (from Fig. 2) is also processed by a Fourier-based high pass filter with a stopband frequency of 150 Hz and passband frequency of 250 Hz. The high pass filtered results are shown on Row 2 in Fig. 4. Similarly, the filtered signal is convolved with a Hanning window of 0.05 sec (Fig. 4, Row 3) and computed by the autocorrelation method (Eq. 2) to get the periodicity information (Fig. 4, Row 4). The periodicity information of high pass filtering along with the derived heart rate (Fig. 2) and rolling ball sifting results (Fig. 3) are combined to form an augmented visual inspection system presented in Fig. 5a.

### Augmented Visual Inspection

Figure 5 illustrates 4 example cases of our proposed augmented visual inspection system for the identification of carotid bruits. The cardiac cycle, extracted bruits, and their periodicity information presented in Figs 2, 3, 4 are merged and aligned in Fig. 5a accordingly to assist clinicians in making a diagnosis based on three rules. First, heart rate (HR) is detectable (marked as HR (+) in Table 1) if pulses on Row 2 in Fig. 5a have distinct shapes and the heart rate by autocorrelation ranges between 50 and 100 Hz. Second, rolling ball sifting (RBS) bruits appears on Row 3 and their periodicity by autocorrelation (blue curve on Row 5) agrees with HR (marked as RBS (+) in Table 1). Third, high pass filtering (HPF) bruits appear on Row 4 and their periodicity by autocorrelation (green curve on Row 5) agrees with HR (marked as HPF (+) in Table 1). Therefore, Fig. 5a is a positive case since all three rules are satisfied. This case is from the right carotid of a 72 year-of-age male patient with an occlusion level 70–99%. All of the three autocorrelation results agree with a peak value at 77 beats/min as shown on Row 5 in Fig. 5a. This case is categorized as HR (+), RBS (+), HPF (+) in Table 1.

Figure 5b illustrates another example from the left carotid of a 64 year-of-age male patient with an occlusion level 70–99%. The HPF bruits are continuous without a visually recognizable pattern. The autocorrelation method also fails to generate a peak value at 74 beats/min as compared with the HR results. However, the rolling ball sifting algorithm is able to discriminate lower frequency waves to generate a bruit pattern on Row 3 with an autocorrelation peak at 74 beats/min on Row 5 in Fig. 5b. This case is categorized as HR (+), RBS (+), HPF (−) in Table 1.

Figure 5c illustrates an example from the right carotid of a 54 year-of-age female patient with an occlusion level less than 50%. The HPF bruit pattern and its autocorrelation result indicate that the detected bruits are very likely to be true. We review the same dataset in the diaphragm mode and confirm that the faint bruits indeed are detectable by the rolling ball sifting algorithm before they are smoothed by the bell mode filter provided by the digital stethoscope’s processing software system. This case is categorized as HR (+), RBS (−), HPF(+) in Table 1.

Last, Fig. 5d illustrates the only 100% occlusion case in the whole dataset of 96 carotid arteries. This case is particularly interesting because most completely occluded carotid arteries do not generate audible sounds because blood no longer flows through the occluded arterial lumen. However, this case from the right carotid artery of a 85 year-of-age male patient shows a clear periodical pattern in all three indicators with their autocorrelation peaks around 43 beats/min. In order to rule out that such a low heart rate (<50 Hz) can be possibly caused by other factors, the patient’s left carotid artery with an occlusion level 50–70% is also reviewed. The autocorrelation peaks derived the left carotid are also 43 beats/min. This case is therefore categorized as HR (+), RBS (+), HPF (+) in Table 1.

### Statistical Analysis

In summary, 83 (86.5%) out of 96 carotid arteries are successfully examined with measurable heart rate (satisfying the first rule). Among these 83 cases, 28 (33.7%) have detectable bruits which satisfy at least one of the second and third rules (RBS (+) or HPF (+)). The rates of detectable bruits are 10% (2/20) for the cases that are absence of atherosclerotic plaque, 34.4% (11/32) for low risk stenosis cases (occlusion <50% and 50–70%), and 46.7% (14/30) for high risk stenosis cases (occulsion 70–99%). The details are summarized in Table 1.

The presence or absence of atherosclerotic plaque as defined by computed tomography angiography (CTA) with bruit detection results are listed in Table 2. Based on 83 HR(+) cases, the sensitivity, specificity, positive predictive value (PPV), and negative predictive value (NPV) for predicting the presence (63 cases) or absence (20 cases) of atherosclerotic plaque are 41.3% (26/63) (95% confidence interval (CI): 29.0% to 54.4%), 90% (18/20) (95% CI: 68.3% to 98.8%), 92.9% (26/28) (95% CI: 76.5% to 99.1%), and 32.7% (18/55) (95% CI: 20.7% to 46.7%) respectively. Their confidence intervals are “exact” Clopper-Pearson confidence intervals.

## Discussion

Here we discuss the results in Tables 1 and 2 and Fig. 5. First, rolling ball sifting by a large radius successfully detect the heart rate in 83 cases with a successful rate of 86.5%. This process is fully automatic with limited human interference except choosing the signal segment for estimation. The resultant heart sound pattern is visually available in the proposed augmented visual inspection system as shown in Fig. 5 to help clinicians in identifying systolic cycles. The heart rate computed by using the autocorrelation method provides a quantitative evaluation of bruit periodicity derived from both rolling ball sifting and high pass filtering approaches. We also investigate the causes of the failed 13 cases and find that 2 cases are caused by heavy breathing, 5 cases are caused by loud noisy spikes, and 6 cases are caused by unknown reasons. Therefore, 7 out of 13 failed cases are possibly to be improved if the user is allowed to record the carotid sound again.

Second, the high pass filtering and rolling ball sifting results (HPF and RBS) agree in 76 (91.6%) out of 83 HR (+) cases. Cohen’s unweighted kappa (*κ*) between HPF and RBS is 0.797 for these 83 HR (+) cases. A rule of thumb is that a *κ* of 0.70 or above indicates adequate interrater agreement. As a result, we find that diagnostic decisions are made rather quickly with high confidence for the agreement cases because both qualitative and quantitative periodicity information is presented to the user visually. Among the 7 disagreement cases, the cases such as Fig. 5b,c all suggest that the quality of periodicity information of either RBS (+) in Fig. 5b or HPF (+) in Fig. 5c alone is an effective bruit detector. Therefore, rolling ball sifting and high pass seem to complement each other in these disagreement cases.

Third, we found that specificity (90%) and PPV (92.9%) of diagnosing atherosclerosis by the existence of a bruit detected by using the proposed method were very high as opposed to the high false positive rates commonly described in textbooks. One possible explanation is that the proposed periodicity tests eliminate most random noise patterns extracted by the traditional high pass filtering approach. Another possible explanation is that CTA is more sensitive in detecting small atherosclerotic plaques than ultrasound as ultrasound is also known to be operator-dependent. On the other hand, the resultant sensitivity (41.3%) and NPV (32.7%) were low and rather similar to other studies. Therefore, the causal relationship between the fluid dynamics and bruits/plaque formation requires further studies to investigate why some stenoses cause bruits and others do not.

Please note that no clinicians’ findings are used as the gold standard in this study because our protocol allows patients to breath quietly during carotid sound recording. The existence of breathing sound tends to interfere human perception to some degree. However, the high *κ* value (0.797) between the rolling ball sifting and high pass filtering methods suggests that computer-aided auscultation can be done consistently. Furthermore, in a real clinical setting where patients are unable to hold their breath, breathing quietly for more than 5 seconds may be a plausible alternative protocol for carotid auscultation with the help of the proposed visual inspection system.

## Methods

### Materials

48 patients with their CTA examinations performed in our hospital were recruited to participate in this Institutional Review Board approved study. All experimental protocols were approved by the Ethics Committee of the National Taiwan University Hospital and adhered to the tenets of the Declaration of Helsinki. Written informed consent was obtained from all subjects and the methods were carried out in accordance with the relevant guidelines and regulations. The sounds of both the right and left carotid arteries were recorded by an electronic stethoscope (3M Littmann Model 3200, Minnesota, USA) with the recommended bell mode setting and digitized with sampling rate 4000 Hz. The sound collection length was between 5.6–30 seconds (mean value 15.6 +/− 6.3) and the patients were instructed to breathe quietly for their comfort. The bell mode was mainly used to reduce breathing sound. All the diagnostic reports of stenosis levels were reviewed by a board certified radiologist (H.M.L. with over 30 years experience). The stenosis levels and CTA atherosclerotic plaque findings associated with these 96 carotid arteries are summarized in Tables 1 and 2.

### Rolling Ball Sifting Algorithm

In a nutshell, the rolling ball sifting algorithm works by rolling two balls (one above and one below, Fig. 1b) of a suitable radius that is large enough to roll over bruits and yet small enough to ride on low-frequency heart sound waveforms. The raw rolling envelopes (red and green lines in Fig. 1b) are well-defined in the field of computational geometry as alpha shapes and can be found very efficiently by DT. A partial DT is given in Fig. 1c. From the radius of the circumcircle for each DT triangle above the data curve, we derive the maximal upper tangent radius information as


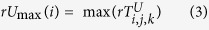


where *U*, *r*, and *T* stand for upper, radius, and triangle respectively; 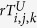 stands for the radius of the circumcircle of the DT triangle of sample points *i*, *j*, and *k* above the data curve. The maximal lower tangent radius can be derived similarly as


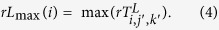


The upper and lower alpha shape envelopes are denoted as *U*_*α*_ abd *L*_*α*_. They are defined as sets of data points (*i*, *x*(*i*)) as


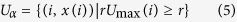


and


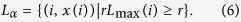


Eqs 5 and 6 may miss faint bruit signals due to the nature of the radius computation for low amplitude signals. The smaller the amplitude is, the flatter the signal may appear. The circles on the right in Fig. 1c show how the radii of tangent circles at the signal troughs increase along the diminishing amplitude value. When the amplitude becomes smaller, *rU*_max_ and *rL*_max_ will appear to be larger and, therefore, the local extrema of faint bruits will be mislabeled as low-frequency components in the regions of overlaid envelopes (thick black lines in Fig. 1b). In order to recover the lost local extrema of faint signals, two different alpha shape envelopes 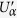 and 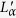 are proposed with additional criteria:


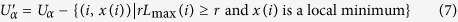



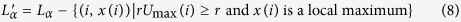


where the second part with ‘−’ recovers the local minima touched by the lower rolling ball in Eq. 7. Similarly, the second part in Eq. 8 recovers the local maxima touched by the upper rolling ball. These ball touched extrema are further processed so that bruit candidate segments with a similar oscillating time scale are merged and inflated to form smoother, separated upper and lower envelopes while low-frequency segments’ envelopes are overlaid together as shown in Fig. 1d. Last, the high-frequency bruits can be extracted by subtracting the mean of the upper and lower envelopes as shown in Fig. 1d,e.

## Additional Information

**How to cite this article**: Huang, A. *et al.* Rolling ball sifting algorithm for the augmented visual inspection of carotid bruit auscultation. *Sci. Rep.* **6**, 30179; doi: 10.1038/srep30179 (2016).

## Supplementary Material

Supplementary Information

## Figures and Tables

**Table 1:** Stenosis levels and bruits associated with the test dataset of 96 carotid arteries.

Periodicity			Degree of Occlusion					subtotal
HR	RBS	HPF	absence	<50%	50–70%	70–99%	100%	
+	+	+	1	3	4	12	1	21
+	+	−	1	1	0	1	0	3
+	−	+	0	3	0	1	0	4
+	−	−	18	17	4	16	0	55
−			7	2	0	4	0	13
Total			27	26	8	34	1	96

HR, RBS, and HPF represent detectable heart rate, rolling ball sifted bruits, and high pass filtered bruits, respectively.

**Table 2:** The presence and absence of atherosclerotic plaque in carotid CT angiography and associated bruit detection of 96 tested carotid arteries.

Periodicity			Atherosclerotic Plaque		subtotal
HR	RBS	HPF	absence	presence	
+	+	+	1	20	21
+	+	−	1	2	3
+	−	+	0	4	4
+	−	−	18	37	55
−			7	6	13
Total			27	69	96

HR, RBS, and HPF represent detectable heart rate, rolling ball sifted bruits, and high pass filtered bruits, respectively.
