# Supplementary material for: Rolling ball sifting algorithm for the augmented visual inspection of carotid bruit auscultation
Source: Sci Rep. 2016 Jul 25;6:30179. doi: 10.1038/srep30179 (PMC4958970; doi:10.1038/srep30179)
Supplement: Supplementary Information [file srep30179-s1.pdf]

Supplementary Information

Rolling ball sifting algorithm for the augmented visual inspection of carotid bruit auscultation

Adam Huang[1], Chung-Wei Lee[2], Hon-Man Liu[2,3,*]

[1]Research Center for Adaptive Data Analysis, National Central University, Jhongli, 32001, Taiwan
[2]Department of Medical Imaging, National Taiwan University Hospital, Taipei, 100, Taiwan
[3]Department of Radiology, College of Medicine, National Taiwan University, Taipei, 100, Taiwan
*Corresponding author: hmliu@ntu.edu.tw

```
function DrawFig5(x,y,Fs)
% Supplementary Information for the manuscript
% "Rolling Ball Sifting Algorithm for the
% Augmented Visual Inspection of Carotid Bruit Auscultation"
% by Adam Huang, Chung-Wei Lee, Hon-Man Liu* (hmliu@ntu.edu.tw)
% submitted to Scientific Reports
% input: x input signal's time
%        y input signal's value
%        Fs sample frequency
% output: augmented visual inspection system as shown in Fig. 5
% code author: Adam Huang, RCADA, National Central University
% email: adamhuan@gmail.com
% date: 2016/04/08

figure,
% % Row1 original carotid sound signal
subplot(5,1,1); % plot the input signal on Row 1
plot(x,y,'k');axis tight;
% % Row2 heart sound pattern
% compute the rolling ball sifting (RBS) envelopes by a large radius
% cutoff frequency 5 Hz, rescale factor 50
[~,~,upper,lower]=rcada_rbsift_1(y,Fs,5,50,0);
HB=upper-lower; % extracted heart sound pattern
subplot(5,1,2); % plot the RBS heart sound pattern on Row 2
plot(x,HB,'r');axis tight;
% % Row3 rolling ball sifting bruits
% compute the RBS envelopes by a small radius
% cutoff frequency 200 Hz, rescale factor 50
IMF1=emd_imf1(y); % extract IMF1 first by the EMD alg.
[H200,~,upper,lower]=rcada_rbsift_1(IMF1,Fs,200,50,0);
B=upper-lower;% envelope of bruit > 200 Hz
subplot(5,1,3); % plot RBS bruits
plot(x,H200,'b');axis tight;
% % Row4 high pass filtering bruits
% compute high pass filtering (HPF) bruits
% stopband 150 Hz, passband 250 Hz
d = designfilt('highpassiir','StopbandFrequency',150 ,...
  'PassbandFrequency',250,'StopbandAttenuation',65, ...
  'PassbandRipple',0.5,'SampleRate',Fs,'DesignMethod','butter');
HP200=filter(d,y);
subplot(5,1,4); % plot the HPF bruits on Row 4
plot(x,HP200,'g');axis tight;
% % Row5 periodicity by autocorrelation
[HR]=xcorr(HB,3*Fs,'coeff'); % heart rate by autocorrelation of HB
% convolve the RBS bruits' envelope diff by a Hanning window of 0.05
 sec
RB=conv(B,hann(Fs*0.05),'same');
[RBS]=xcorr(RB,3*Fs,'coeff'); % RBS bruits periodicity
% convolve the HPF bruits by a Hanning window of 0.05 sec
RHP=conv(abs(HP200),hann(Fs*0.05),'same');
[HPF]=xcorr(RHP,3*Fs,'coeff'); % HPF bruits periodicity
h=subplot(5,1,5); % plot the periodicity of HR, RBS, HPF
```

```
ix=1:length(HR);
plot(ix,HR,'r',ix,RBS,'b',ix,HPF,'g');axis tight;
h.XTick=     [0.7*Fs      ,1.8*Fs,2.2*Fs,3*Fs,3.8*Fs,5.2*Fs];
h.XTickLabel={'Heart rate','50'  ,'75'  ,'x' ,'75'  ,'Beats/min'};

function [H,L,upper,lower]=rcada_rbsift_1(y,fs,ft,s,flag)
% rolling ball sifting algorithm
% input: y signal
%        fs signal sample frequency
%        ft threshold (cutoff) frequency
%        s re-scale factor y=s*y
%        flag>0 to draw results
% output: H high frequency component
%         L low frequency component
%         upper envelope
%         lower envelope
% author: Adam Huang, RCADA, National Central University
% email: adamhuan@gmail.com
% date: 2016/03/17

% % arrange data as a column (m by 1) vector
[nrow,ncol]=size(y);
if nrow<ncol
   y=y';
end
r=fs/ft/4;% Eq. (1), R-ball's radius for frequency sifting threshold:
 Ft
% % step 0--find the radius info rU and rL by Delaunay triangulation
% preprocessing to rescale/resample data, find touching ball's radius
 info
% rU & rL (Eqs. 2&3) for upper envelope and lower envelope
x=(1:length(y))';
[rU,rL]=rcada_rb_init(x,y,s);% apply Delaunay triangulation
% % step 1--find alpha-shape envelopes by rolling ball touched points
iU1=(rU>=r); % upper envelope points touched by upper ball radius r
iL1=(rL>=r); % lower envelope points touched by lower ball radius r
% first and last points are also marked as "touched"
iU1(1)=1;iL1(1)=1;iU1(end)=1;iL1(end)=1;
% % step 2--inflate and form initial intermittent signal segments
iU2=iU1;iL2=iL1; % updated point touching info
% local max and min points
iMX=((y-[y(1);y(1:end-1)]>=0) & (y-[y(2:end);y(end)]> 0)) |...
    ((y-[y(1);y(1:end-1)]> 0) & (y-[y(2:end);y(end)]>=0));
iMN=((y-[y(1);y(1:end-1)]<=0) & (y-[y(2:end);y(end)]< 0)) |...
    ((y-[y(1);y(1:end-1)]< 0) & (y-[y(2:end);y(end)]<=0));
ii=(iU1>iL1); % points touched by upper R-ball but not lower one
jj=ii & iMX; % plus condition: local maxima
[sg0,sg1]=findSegments(ii); % segments touched by upper ball only
for i=1:length(sg0) % only keep the local max touched by upper-ball
    iU2(sg0(i):sg1(i))=jj(sg0(i):sg1(i));
end
ii=(iL1>iU1); % points touched by lower R-ball but not upper one
jj=ii & iMN; % plus condition: local minima
[sg0,sg1]=findSegments(ii);% segments touched by lower ball only
```

```
for i=1:length(sg0) % only keep the local min touched by lower-ball
    iL2(sg0(i):sg1(i))=jj(sg0(i):sg1(i));
end
iL2(1)=1;iL2(end)=1;iU2(1)=1;iU2(end)=1;
% % step 3 recover faint local extrema
iU3=iU2;iL3=iL2; % updated point touching info
iL3(iU2>0 & iMX)=0;... % Eq. (2) % recover touched maxima
iU3(iL2>0 & iMN)=0;... % Eq. (3) % recover touched minima
iL3(1)=1;iL3(end)=1;iU3(1)=1;iU3(end)=1;
% % step 4-- merge fragmental segments
iU4=iU3;iL4=iL3;  % updated point touching info
pflag=0;% peak flag
tflag=0;% trough flag
for i=2:length(y)-1
    if iU3(i)>0 && iL3(i)<1 % envelope local peak
        ipeak=i;
        pflag=1;
    elseif iU3(i)<1 && iL3(i)>0 % envelope local trough
        itrou=i;
        tflag=1;
    end
    if pflag>0 && tflag>0 % merge any peak-trough segment <= 2*r+2
        if itrou>ipeak
            pflag=0;
            if x(itrou)-x(ipeak)<=2*r+0.5 % allows some peak-trough
 error
                iU4(ipeak+1:itrou-1)=0;
                iL4(ipeak+1:itrou-1)=0;
            end
        else
            tflag=0;
            if x(ipeak)-x(itrou)<=2*r+0.5 % allows some peak-trough
 error
                iU4(itrou+1:ipeak-1)=0;
                iL4(itrou+1:ipeak-1)=0;
            end
        end
    end
end
% % stap 5--remove single peaks and troughs
% examine every air inflated segment and count its peaks and troughs
% remove stand-alone peaks and trough
iU5=iU4;iL5=iL4; % updated point touching info
fflag=0;
npeak=0;
ntrou=0;
for i=2:length(y)-1
    switch (fflag)
        case 0
            if iU4(i)<1 || iL4(i)<1 % an inflated segment starts
                b0=i;
                fflag=1;
            end
        case 1
```

```
                if iU4(i)>0 && iL4(i)>0 % an inflated segment end
                    fflag=0;
                    if npeak<1 || ntrou<1 % stand alone peak or trough
                        iU5(b0:i-1)=1;
                        iL5(b0:i-1)=1;
                    end
                    npeak=0; % reset peak counter
                    ntrou=0; % reset trough counter
                end
                if iU4(i)>0 && iL4(i)<1 % peak
                    npeak=npeak+1;
                end
                if iL4(i)>0 && iU4(i)<1 % trough
                    ntrou=ntrou+1;
                end
        end
end
% % step 6 compute upper/lower envelopes and derive high/low-freq
 components
upper=envlp(x,y,find(iU5>0));% rolling ball upper envelope
lower=envlp(x,y,find(iL5>0));% rolling ball lower envelope
L=(upper+lower)/2;%low-freq component as the mean of upper&lower
 envelopes
H=y-L;% high-freq component
% % draw results if flag is set
if flag>0
    mx=max(y(:));
    figure;
    hold on;%original data
    drawResults(x,y-2*mx,iU1,iL1);%initial alpha-shape envelopes
    drawResults(x,y-4*mx,iU2,iL2);%inflate
    drawResults(x,y-6*mx,iU4,iL4);%recover faint signals and merge
    drawResults(x,y-8*mx,iU5,iL5);%remove stand alone peaks/troughs
    plot(x,y-10*mx,'k',x,H-10*mx,'r');%high-freq component
    plot(x,y-12*mx,'k',x,L-12*mx,'g');%low-freq component
    hold off;
end

function [y1]=envlp(x,y,iX)
% find the envelope with spline using pchip
x0=x(iX);
y0=y(iX);
y1=pchip(x0,y0,x);

function [sg0,sg1]=findSegments(ii)
ii=double(ii);
ii0=[0;ii(1:end-1)]-ii;
sg0=find(ii0<0);% strip starting
ii1=ii-[ii(2:end);0];
sg1=find(ii1>0);% strip ending

function [rU,rL]=rcada_rb_init(x0,y0,s)
% find tangibility radius info by Delaunay triangulation
[x,y,ix]=resample(x0,y0,s);% insert extra data points if necessary
```

```
TR=delaunay(x,y);% Delaunay triangulation
% sort triangles (denoted as ABC) so that x-coord: x(A)<x(B)<x(C)
TR=sort(TR,2);
AC=[x(TR(:,3))-x(TR(:,1)) y(TR(:,3))-y(TR(:,1))];% vectors AC
BC=[x(TR(:,3))-x(TR(:,2)) y(TR(:,3))-y(TR(:,2))];% vectors BC
AB=[x(TR(:,2))-x(TR(:,1)) y(TR(:,2))-y(TR(:,1))];% vectors AB
NAC=sqrt(sum(AC.*AC,2));% lengths of side AC
NBC=sqrt(sum(BC.*BC,2));% lengths of side BC
NAB=sqrt(sum(AB.*AB,2));% lengths of side AB
AREA=(AB(:,1).*AC(:,2)-AC(:,1).*AB(:,2))*0.5;% area by cross product
RADI=(NAC.*NBC.*NAB./abs(AREA))*0.25;% circumscribed radius formula
% find rU and rL Eqs. (a) and (b)
rU=zeros(length(x),1);
rL=zeros(length(x),1);
for i=1:size(TR,1)
    if AREA(i)>=0 % triangle is above the curve
        for j=1:3
            rU(TR(i,j))=max(rU(TR(i,j)),RADI(i));
        end
    else % triangle is below the curve
        for j=1:3
            rL(TR(i,j))=max(rL(TR(i,j)),RADI(i));
        end
    end
end
rU=rU(ix); % only return for the original data points
rL=rL(ix);

function [x1,y1,ix]=resample(x,y,s)
% resample data so that distance between neighbor points < 1
len=length(x);
ix=(1:len)';% index to the original data position
y=y*s;% rescale
dx=x(2:len)-x(1:len-1);
dy=y(2:len)-y(1:len-1);
d=floor(((dx.^2+dy.^2).^0.5));% distance between points
% if neighbor points' distance d >= 1, insert d points
len1=len+sum(d);
x1=zeros(len1,1);
% arrange insertion
counter=0;
for i=1:len-1
    counter=counter+1;
    ix(i)=counter;
    x1(counter)=x(i);
    dx=(x(i+1)-x(i))/(d(i)+1);
    for j=1:d(i)
        counter=counter+1;
        x1(counter)=x(i)+j*dx;
    end
end
counter=counter+1;
ix(len)=counter;
x1(counter)=x(len);
```

```
y1=pchip(x,y,x1);% monotone piecewise cubic spline interpolation

function drawResults(x,y,iU,iL)
upper=envlp(x,y,find(iU>0)); % piecewise linear upper envelope
lower=envlp(x,y,find(iL>0)); % piecewise linear lower envelope
plot(x,y,'k','LineWidth',0.5);
plot(x,upper,'k','LineWidth',0.75);
plot(x,lower,'k','LineWidth',0.75);
plot(x,(upper+lower)/2,'k--','LineWidth',0.75);

function [imf1]=emd_imf1(y)
% emd algorithm
% input: y signal
% output: imf1 first imf
% author: Adam Huang, RCADA, National Central University
% email: adamhuan@gmail.com
% date: 2016/03/17
% % arrange data as a column (m by 1) vector
[nrow,ncol]=size(y);
if nrow<ncol
   y=y';
end
dd=(1:length(y))';
for i=1:10
    iMX=((y-[y(1);y(1:end-1)]>=0) & (y-[y(2:end);y(end)]> 0)) |...
        ((y-[y(1);y(1:end-1)]> 0) & (y-[y(2:end);y(end)]>=0));
    iMN=((y-[y(1);y(1:end-1)]<=0) & (y-[y(2:end);y(end)]< 0)) |...
        ((y-[y(1);y(1:end-1)]< 0) & (y-[y(2:end);y(end)]<=0));
    iMX(1)=1;iMX(end)=1;
    iMN(1)=1;iMN(end)=1;
    upper= spline(dd(iMX),y(iMX),dd);
    lower= spline(dd(iMN),y(iMN),dd);
    y=y-(upper + lower)/2;
end
imf1=y;
```

Published with MATLAB® R2015a
